# Peptidomics in Different Phases of Severe Acute Pancreatitis Model

**DOI:** 10.1002/prca.70035

**Published:** 2025-11-29

**Authors:** Roberto Rasslan, Marcia Kiyomi Koike, Marcel Cerqueira Cesar Machado, Luiz Felipe Martucci, Leo Kei Iwai, Rosangela Aparecida dos Santos Eichler, Edivaldo Massazo Utiyama, Emer Suavinho Ferro, Edna Frasson de Souza Montero

**Affiliations:** ^1^ Department of Surgery Faculdade De Medicina da Universidade de São Paulo São Paulo Brazil; ^2^ Department of Internal Medicine Faculdade De Medicina da Universidade de São Paulo São Paulo Brazil; ^3^ Department of Pharmacology Instituto De Ciências Biomédicas Da Universidade de São Paulo São Paulo Brazil; ^4^ Instituto Butantan, Laboratory of Applied Toxinology and Center of Toxins, Immune‐Response and Cell Signaling São Paulo Brazil

**Keywords:** bioinformatics analysis, mass spectrometry (nLC‐MS/MS), pathway analysis, peptidomics, severe acute pancreatitis

## Abstract

Severe acute pancreatitis (SAP) involves dynamic shifts from inflammation to immunosuppression, where peptidomic profile evolution may reveal prognostic biomarkers. Here, the plasma peptidome of rats with taurocholate‐induced SAP at 1, 3, 6, 12, and 24 h compared with controls was characterized using nLC‐MS/MS. Ten peptides derived from eight precursor proteins were differentially regulated across time points. The 12‐h period showed eight differentially regulated peptides, while the 6‐ and 24‐h periods had four differentially regulated peptides, and one peptide was regulated between 1 and 3 h. Peptides derived from alpha‐1‐microglobulin (A1M) increased from 3 h onward, while peptides from actin showed major alterations at 12–24 h, coinciding with peak mortality (46%). Bioinformatic enrichment analyses revealed transient activation of mTOR, JAK/STAT, and cell adhesion pathways at 6 h, followed by bacterial invasion and actin cytoskeleton regulation pathways at later stages. These temporal profiles suggest an early antioxidant response and subsequent structural and infection‐related remodeling. These findings suggest that A1M‐derived peptides could represent potential early indicators of disease severity, although further validation in human clinical settings is required. These findings highlight the plasma peptidome as a promising tool for clinical diagnostics, providing a better understanding of SAP progression and identification of phase‐specific biomarkers in pancreatitis.

**Summary:**

This study reveals dynamic changes in the plasma peptidome during the progression of severe acute pancreatitis in rats. Through the identification of differentially regulated peptides, bioinformatic analysis was performed to define the altered pathways and genes, demonstrating the relationship between peptide alterations and disease progression. The 6‐h time point after pancreatitis induction showed the highest number of signaling terms/pathways that characterize the inflammatory phase of the disease. In subsequent moments, the enrichment of pathways related to infection and the regulation of the actin cytoskeleton at 12‐ and 24‐h post‐pancreatitis induction suggests that this period is associated with the process of bacterial translocation and pancreatic necrosis infection. Therefore, the peptide profile and pathways may have implications for defining prognosis and early diagnosis of infection.

## 1 Introduction

Pancreatic necrosis represents 15% of acute pancreatitis and has a mortality rate of 10% to 20% [1, 2, 3] and is characterized by two distinct phases. The first is represented by the exacerbated activation of the systemic inflammatory response, a consequence of the elevated release of pro‐inflammatory cytokines into the circulation, accountable for 50% of deaths. The second, generally within 2 weeks of evolution, is the infection of pancreatic necrosis, responsible for the other half of the mortality. This complication is the determining prognostic factor in the evolution of severe pancreatitis in the late phase of the disease, occurring in 30% of cases [1, 4].

One of the challenges is to define early on which patients will evolve to the severe form, and within this population, who will develop infection. Some serum markers are already established regarding the development of local complications, with C‐reactive protein and procalcitonin being the most used and accessible [5, 6]. The difficulty lies in identifying who will progress to infected pancreatic necrosis and proposing measures to prevent this process from the beginning.

Plasma contains a wide variety of peptides that play roles in homeostasis and physiological function and are associated with various diseases. Peptides regulate cellular function mediated by interaction with proteins, implicating the regulation of genes, metabolism, and cell signaling. This can also occur through short amino acid sequences derived from peptides (short linear motifs—SLiMs) that regulate the interaction between proteins and are associated with signaling pathways [7]. Characterization of the peptidome profile in severe acute pancreatitis may have implications for defining prognosis, early diagnosis of infection, and development of therapeutic strategies [8, 9, 10]. Although the function of some peptides is already defined, the biological function of the vast majority remains unknown.

Both peptidomics and proteomics allow for an analysis that goes beyond the analyzed pathway. Unlike traditional biochemical assessments, which focus on a specific protein network, peptidomics enables the evaluation of multiple peptides and their precursor proteins. In these analyses, it is possible to study protein–protein interaction networks, deepening the knowledge regarding conventional data through bioinformatics, attributing a biological function to proteins and peptides [11]. Another implication of peptidomics is in the identification of biomarkers in numerous diseases [8].

Therefore, peptidomics constitutes an expanding field used to compare biologically significant macromolecules and their possible alterations during disease states. In acute pancreatitis, Papachristou et al. [12] evaluated 28 patients and observed a difference in the serum proteomic profile between the mild and severe forms of the disease, suggesting it may be a tool for early prognosis definition. Wang et al. [10] analyzed proteomics in an experimental model of severe acute pancreatitis and identified changes in the AMPK, MAPK, and PI3K‐AKT signaling pathways. Williams performed a systematic review of the main experimental studies evaluating proteomics in acute pancreatitis and showed an upregulation of proteins related to stress, inflammation, and cytoskeleton, with a downregulation of proteins involved in metabolism [13]. Through the elucidation of peptide expression and the respective altered pathways, using bioinformatics tools, a more comprehensive understanding of the acute pancreatitis pathogenesis can be achieved [14]. Therefore, identifying the peptide profile and pathways is essential for characterizing the different phases of the disease, with the goal of finding prognostic markers and early diagnosis of infection.

## 2 Materials and Methods

This project was approved by the Research Ethics Committee of the Faculdade de Medicina da Universidade de São Paulo (process number 1775‐52/2022) in compliance with the international guidelines for the care and use of laboratory animals.

### 2.1 Surgical Procedures and Experimental Protocol

Male Wistar rats, weighing 250 to 300 g, were distributed into a reference control group and five groups with acute pancreatitis, according to the time of euthanasia: 1, 3, 6, 12, and 24 h. Each group comprised five animals; those that died due to model mortality were replaced. In this model, a mortality rate of 46% was observed between 12 and 24 h after pancreatitis induction.

The anesthetic protocol employed was ketamine (60 mg/kg) combined with xylazine (10 mg/kg) administered intramuscularly. In the pancreatitis groups, following anesthesia, a median laparotomy was performed, exposing the duodenum. The anti‐mesenteric border was then punctured using a 25‐gauge needle (7/10 inch), followed by bile duct catheterization with a polyethylene catheter (Intracath, PE50, Becton–Dickinson, USA). Subsequently, the hepatic hilum was clamped, followed by infusion of a 1.5% sodium taurocholate solution at a volume of 0.5 mL over 1 min. After catheter removal, the duodenal puncture site was sutured, and the abdominal wall was closed. Animals received analgesia with tramadol at a dose of 5 mg/kg every 8 h. At the time of euthanasia, the anesthetic procedure was repeated, and blood collection was performed. The animals in the reference control group were only anesthetized for blood collection without any prior procedure.

### 2.2 Peptide Extraction

Plasma samples (300 µL) of each group (reference control, 1, 3, 6, 12, 24 h after pancreatitis induction) were transferred to a protein low‐binding tube [15, 16]. Equal volume of 0.5% bovine serum albumin, phosphate‐buffered saline containing 0.05% Tween 20, pH 7.2 (sample buffer) plus two volumes of acetonitrile (to achieve 66% of acetonitrile in the final sample extraction volume) was added to the tubes. After vortexing, samples were incubated at room temperature for 60 min and centrifuged at 12,000 × *g* for 5 min at 4°C. The supernatant was transferred to a clean protein low‐binding tube, and the volume was reduced in a speed vacuum centrifuge (Eppendorf, Hamburg, Germany) for 3 h at 30°C. The semi‐dried pellet was resuspended in 1.5 mL of ultrapure water, acidified with 0.1 M HCl (final concentration of 10 mM), and transferred into pre‐washed Amicon Ultra‐4 Centrifugal Units of 10,000 Da cutoff (Millipore, Burlington, MA, USA). After centrifugation at 1500 × *g*, at 4°C, for approximately 1 h, the pH of the flow through (containing peptides of molecular mass < 10,000 Da) was adjusted to 2–4 with 50% formic acid (Fisher Scientific, Pittsburgh, PA, USA). This flow‐through material containing peptides was passed through C18 Oasis HLB 1cc (30 mg) Extraction Cartridges (Waters, Etten‐Leur, NB, NL) previously equilibrated with 100% acetonitrile/0.10% formic acid (Fisher Scientific, Pittsburgh, PA, USA), and then with 5% acetonitrile/0.10% formic acid in ultrapure water. After washing with 5% acetonitrile/0.10% formic acid, peptides were eluted in 100% acetonitrile/0.15% formic acid, collected in low‐retention microcentrifuge tubes, and dried in a speed vacuum centrifuge (Eppendorf, Hamburg, Germany). The pellet was resuspended in ultrapure water (100 µL) and quantified by 214 nm absorbance (NanoDrop, Thermo Fisher Scientific, São Paulo, Brazil), using a peptide mix of known composition and concentration as the standard reference for determining peptide concentration (standard curve).

### 2.3 Peptide Labeling

A 10 µg of purified peptide extract was diluted in 100 µL of triethylammonium bicarbonate (TEAB) buffer (Sigma‐Aldrich, St. Louis, USA) to a final concentration of 100 mM. In the hood, 4 µL of the different isotopic forms of the formaldehydes were added, according to the desired labeling scheme, to a final concentration of 0.15% [15, 16]. Then, sodium cyanoborohydride (NaBH3CN) was added to a final concentration of 22 mM, and samples were incubated for 16 h at room temperature, protected from light. The reaction was then quenched with 16 µL of 1% ammonium bicarbonate. Samples were placed on ice for 5 min and acidified with 0.4% formic acid (Sigma‐Aldrich, St. Louis, USA). Four differentially labeled samples were pooled (allowing every run to have all four markers included), desalted using Oasis HLB 1cc (30 mg) Extraction Cartridges (Waters, Etten‐Leur, NB, NL), and eluted with 100% acetonitrile containing 0.15% formic acid. The samples were passed into detergent removal spin columns, following instructions according to the manufacturer (Thermo Fisher Scientific,Rockford, IL, USA), eluted with 100 uL of ammonium bicarbonate 100 mM, dried in a vacuum centrifuge and stored at −20°C until use. The amount of 0.5 µg of peptides diluted in 5 µL of 100% acetonitrile containing 0.15% formic acid was injected for all mass spectrometry analyses, as described below.

### 2.4 Mass Spectrometry Analysis

Mass spectrometry peptidome analyses were performed on an Orbitrap Exploris 480 Spectrometer (Thermo Fisher Scientific, Bremen, Germany) coupled to a Vanquish Neo nanoLC liquid chromatographer (Thermo Fisher Scientific) with an Easy Flex ion source Peptide separation was performed on a nanoLC Acclaim PepMap NEO analytical column (150 mm x 75 µm, 2 µm) and a nanoLC Acclaim PepMap 100 C18 trap column (20 mm x 75 µm, 2 µm) (Thermo Fisher Scientific). Peptides were eluted using a linear gradient of 5%–30% solvent B (acetonitrile, in 0.1% formic acid) for 75 min and 30%–40% solvent B for 7 min at 300 nL/min flow. The mass spectrometer, equipped with a FAIMS (field asymmetric ion mobility spectrometry) system, performed gas‐phase ion separation based on their different mobilities under alternating electric fields. Data were acquired after the generation of multiple peptides protonated by the ESI (electrospray ionization), according to the following conditions. Runtime, 90 min, polarity positive, default charge state 2, full MS, resolution 60,000, maximum IT 50 ms, scan range 400 to 1000 m/z. AGC target: 300% for Full MS and 1000% for fragment spectra, maximum IT 50 ms, isolation window 2 m/z. The normalized collision energy was set to 28% and 32%. MS/MS spectra were acquired with FAIMS compensation voltages set to −45 and −60 V, and a resolution of 30,000. Data processing and analyses were performed *in‐house* using XCalibur and the Mascot software suite, as previously described [15, 16]. Thus, to perform data analyses, raw data files were converted into peak list format (mgf) by Mascot Daemon (v.2.3 Matrix Science Ltd., London, UK, search engine v.2.8). No cleavage site was specified, and a fragment ion mass tolerance of ±.5 Da was applied to the MS and MS/MS ions. The search parameters were no enzyme specificity, precursor mass tolerance set to ±0.5 Da; no modifications included. The identified peptides were filtered to keep only those in which the ratio value has shown 2 or higher of increase, or 0.5 or lower of decrease compared to the reference group.

### 2.5 Bioinformatics and Statistical Analysis

In mass spectrometry, the peptide ratio value between groups indicates its increase or a decrease. We evaluated whether the peptide ratios of animals submitted to the acute pancreatitis protocol at different times of blood plasma collection relative to the control group were significantly different from the null effect of 1. For this, we used the non‐parametric Wilcoxon signed rank test for one sample. *p* values ≤ 0.05 were considered statistically significant. All analyses were performed using R (v.4.1).

Active subnetwork identification within a protein interaction network (PIN) and subsequent enrichment analyses were performed using pathfindR (v.1.6.4) [17] with genes encoding the source protein of differentially regulated peptides as input. The Search Tool for the Retrieval of Interacting Genes/Proteins (STRING) was selected as the reference PIN, and the Kyoto Encyclopedia of Genes and Genomes (KEGG) was used as the reference gene set [16]. Short Linear Motifs (SLiMs) of differentially regulated peptides were identified using the Eukaryotic Linear Motif (ELM) prediction tool [18]. Only SLiMs with annotated Mus musculus instances from docking or ligand classes were included for the analyses. Enrichment of the SLiMs selected by this process was assessed by calculating their overrepresentation (ORA) per KEGG term, using Fisher's exact test. The reference set for these analyses was the ELM database of SLiMs for KEGG terms of Mus musculus. A *p* value ≤ 0.05 was considered significant. The odds ratio was assumed as the representative value of the magnitude of enrichment [16].

## 3 Results

**FIGURE 1:**
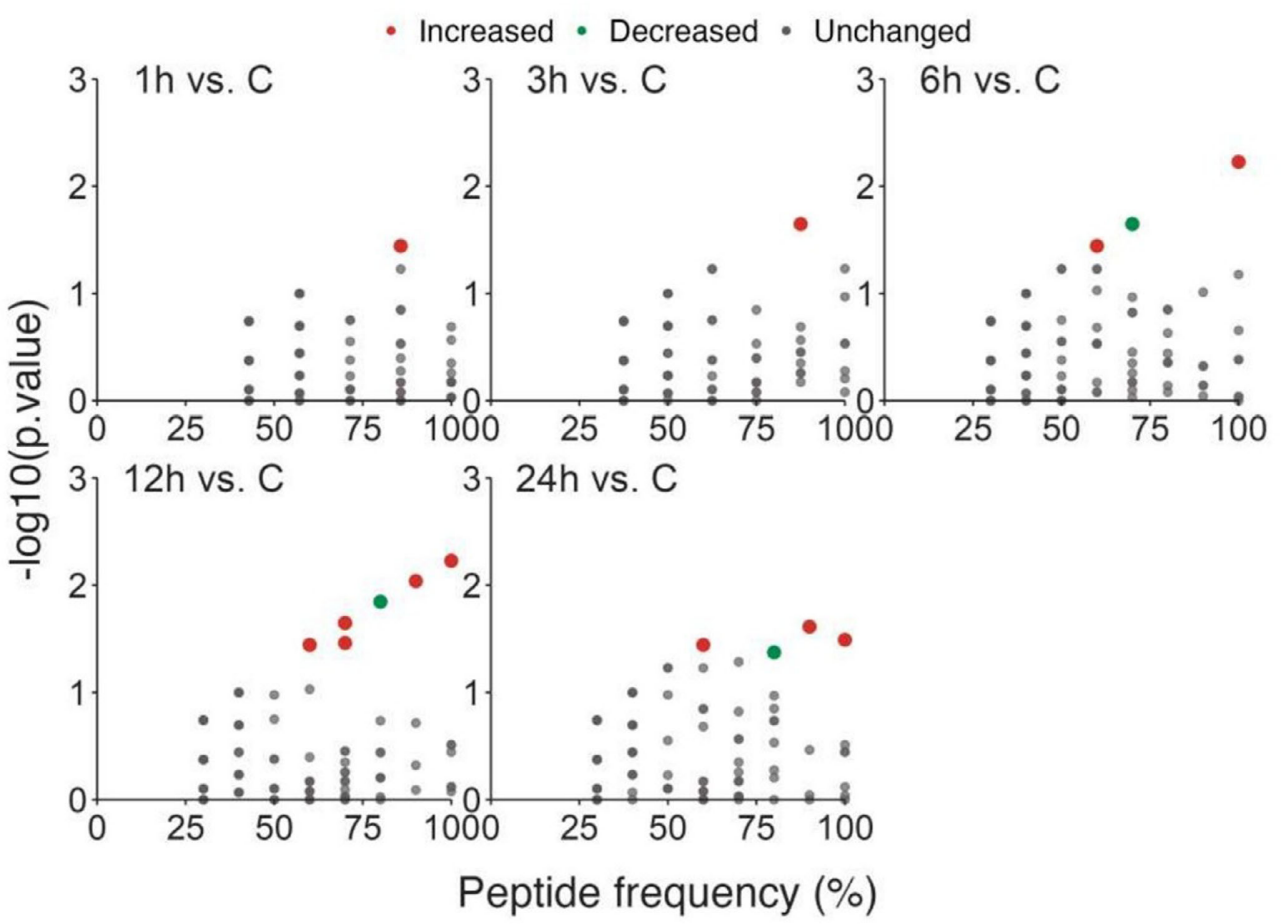
Plasma peptides frequency and their respective *p* value when comparing peptide ratios of 1 h, 3, 6, 12 and 24 h vs. control. Colored dots represent peptides whose relative ratio either remained unaltered (gray dots), significantly increased (red dots), or significantly decreased (green dots) [*p* value ≤ −log10 (5·10−2)].

Ten peptides originating from eight distinctive proteins were observed herein, and all were differentially regulated at some point (1–24 h) compared to the control group (Table 1; Figure 1); importantly, only peptides showing statistically significant differential regulation (*p* ≤ 0.05) compared to the control group were included in Table 1. Alpha‐1‐microglobulin (A1M) generated the following peptides, SAPFSSDSEQGNA, AYLTSASSRPT, APFSSDSEQGNA, with an average frequency of 100%, 73% and 66%, respectively, among the groups. Two peptides derived from actin cytoskeleton proteins were identified at 12 and 24 h post pancreatitis induction, with one increased and the other decreased compared to the control group. The peptide RKEEPPSLRPAPPPISGGGY, derived from fibrinogen, was the only one that increased early. Two other peptides derived from fibrinogen showed high frequency, but without statistical difference in relation to the control group (Table 1; Figure 1).

**TABLE 1:** Peptide frequency and group comparisons.

	Freq. (%)^a^	1H vs. C	3H vs. C	6H vs. C	12H vs. C	24H vs. C
**A1M**						
SAPFSSDSEQGNA	100 ± 0	↔	↔	↑	↑	↑
AYLTSASSRPT	73.79 ± 7.69	↔	↑	↑	↑	↔
APFSSDSEQGNA	66.79 ± 5.16	↔	↔	↑	↑	↔
**IC1**						
SSQDPLVVQEGSR	73.64 ± 9.18	↔	↔	↔	↑	↔
**MYL1**						
AAPAPAPAPAPAPAKPKEE	71.93 ± 16.61	↔	↔	↔	↑	↑
**FIBB**						
RKEEPPSLRPAPPPISGGGY	68.64 ± 16.91	↑	↔	↔	↔	↔
**ACTS**						
VAPEEHPTLLTEAPLNPK	59.29 ± 1.43	↔	↔	↔	↑	↑
**ACTG**						
VAPEEHPVLLTEAPLNPK	58.07 ± 20.5	↔	↔	↔	↓	↓
**HBB1**						
VNPDDVGGEALG	52.57 ± 7.38	↔	↔	↔	↑	↔
**CO4**						
DDPSVHSQPVTPLQLFEGRRS	47.43 ± 17.45	↔	↔	↓	↔	↔

*Note:* C, control animals; 1 h/3 h/6 h/12 h/24 h, after pancreatitis induction procedure.

^a^ Percentage of mass spectrometry runs averaged by groups whose peptide sequence was identified ± standard deviation of frequency across groups. Symbols indicate: ⟷no change in peptide ratio, *p* > 0.05; ↑ or ↓, respectively, peptide ratio either increased or decreased, *p* ≤ 0.05.

Figure 1 shows the number of differentially regulated peptides relative to the control group at various time points after pancreatitis induction. The 12‐h period shows the highest number of eight differentially regulated peptides (Figure 1). The 6‐ and 24‐h periods have four differentially regulated peptides, while after 1 and 3 h, only one peptide was observed.

**FIGURE 2:**
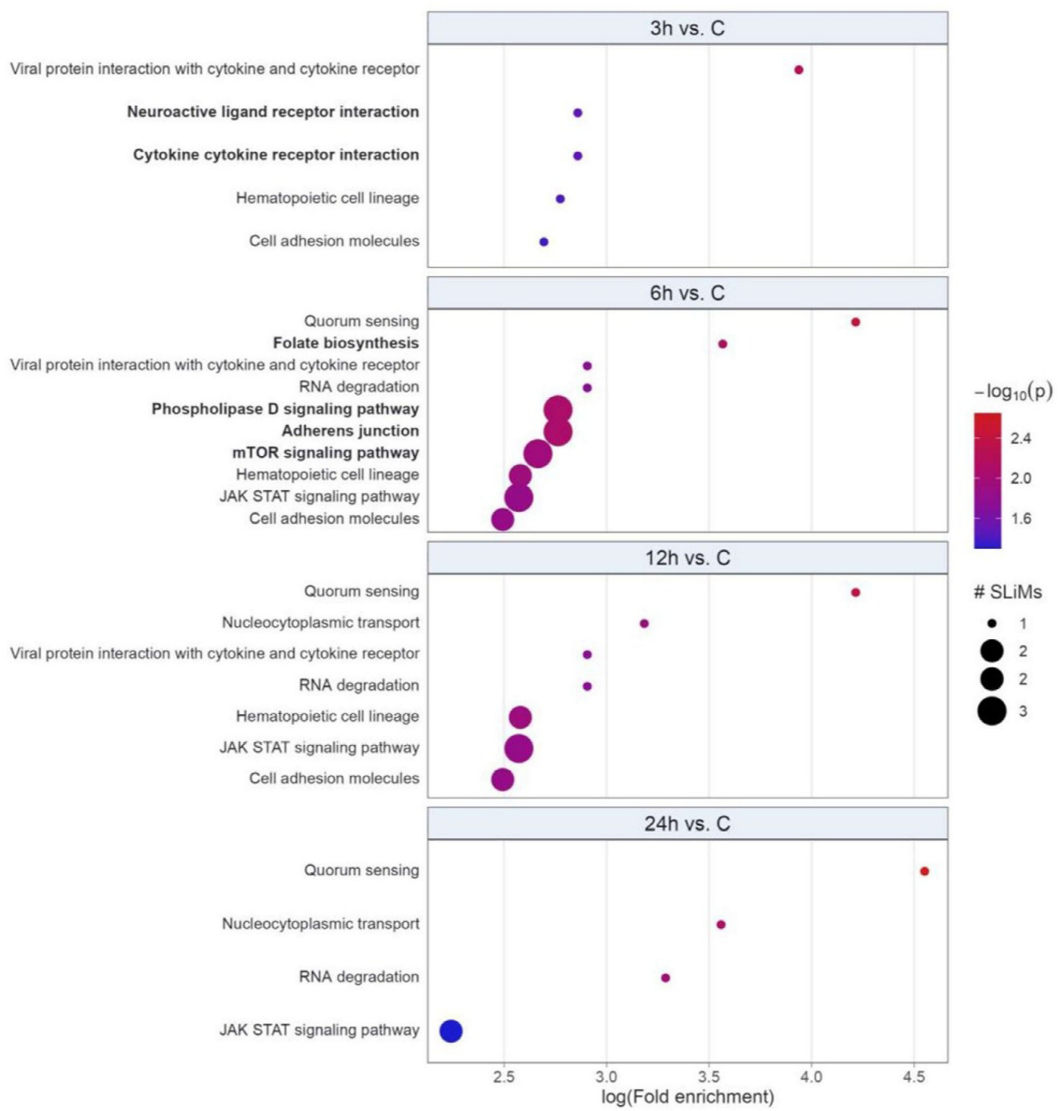
Bubble plot of overrepresented differentially regulated peptides docking or SLiMs KEGG terms. On the *y‐*axis are terms with *p* value ≤ −log10(*p* value) and part of the top 5 lowest *p* values of at least one group comparison (3, 6, 12, or 24 h vs. control Bold terms were enriched in groups exclusively in groups 3 or 6 h vs. control. Bubble size represents number of SLiMs related to a given term. Color scale indicates −log10(*p* value); redder the color, more significantly the term was enriched.

SLiMs are fragments of peptides that have an important role in protein‐protein interaction. They (SLiMs) are protein‐binding modules that play major roles in almost all cellular processes and are central signaling organizers to many cellular pathways. The relationship is complex because each SLiM can interact with a variety of partner proteins, meaning that a single SLiM can influence multiple pathways; SliMs are often highly degenerate. This multi‐functionality allows for dynamic and intricate regulation of many distinctive cell processes. Figure 2 illustrates the enrichment of SLiMs through over‐representation by KEGG terms. The evaluation of pathways through SLiM enrichment was greater after 6 h of pancreatitis induction. During this period, 10 enriched pathways were observed, including mTOR, quorum sensing, JAK/STAT, adherens junctions, cell adhesion molecules, and phospholipase D. The cell adhesion molecules pathway is enriched from 1 h after pancreatitis induction up to 6 h (Figure 2). The quorum‐sensing pathway is enriched at 6, 12, and 24 h after pancreatitis induction, with increasing enrichment in the later phases compared to the control (Figure 2). The JAK/STAT pathway, which is also enriched starting at 6 h, shows decreased enrichment compared to the control group as pancreatitis progresses (Figure 2).

**FIGURE 3:**
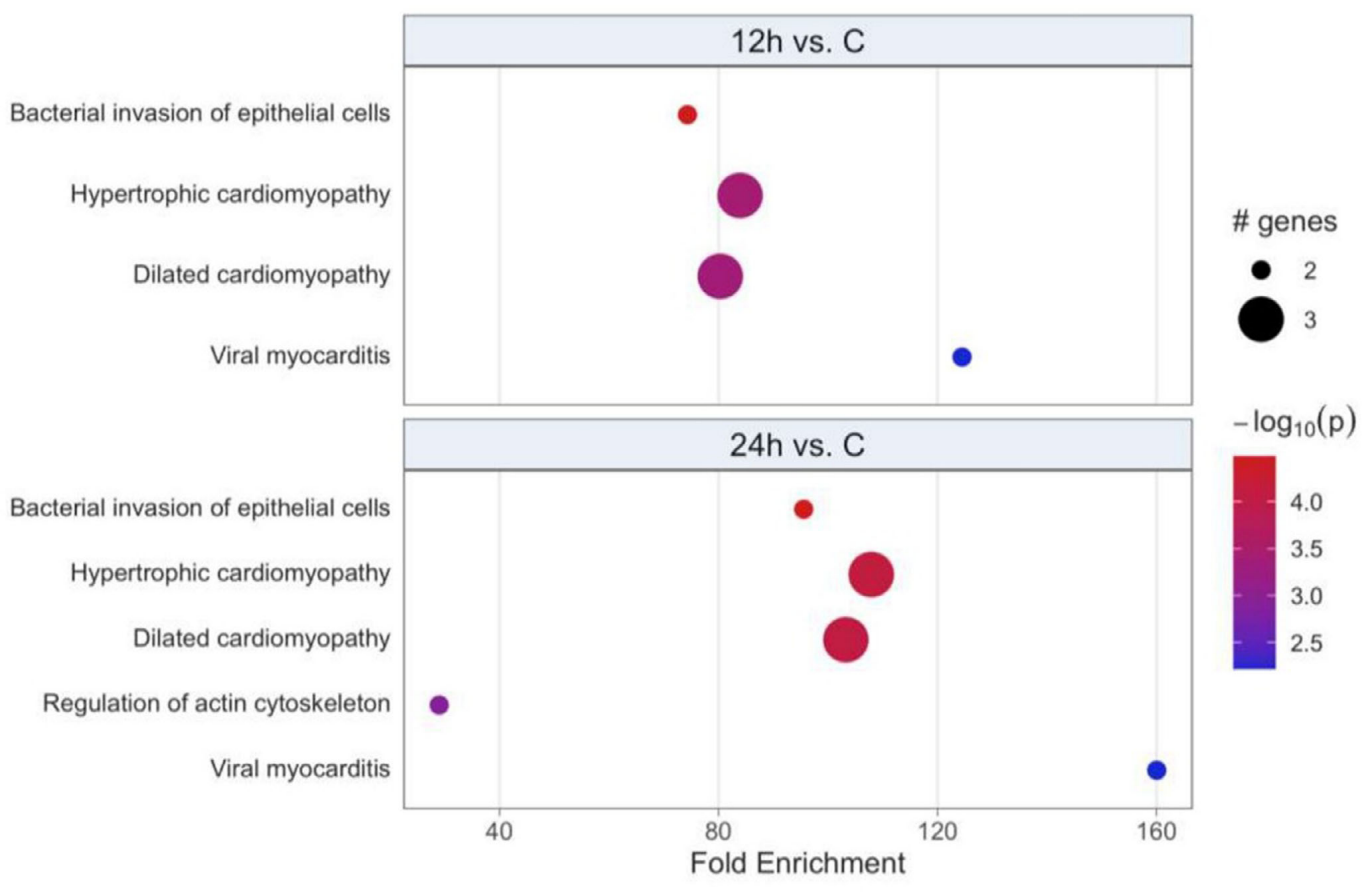
PathfindR bubble plot of biogrid protein interaction network (PIN) represented by kyoto encyclopedia of genes and genomes (KEGG) terms. Enriched PINs using genes encoding the proteins that originated the differentially expressed peptides were identified only for 12 and 24 h vs. control. The *x*‐axis corresponds to fold enrichment values, while the *y*‐axis indicates the enriched pathways with at least two genes. Bubble size indicates number of differentially expressed genes (DEGs) in the given pathway. Color indicates −log10(*p* value) value; the more it shifts from purple to red, the more significant the PIN was enriched.

Protein‐protein interaction networks were constructed using genes encoding the proteins, from which the differentially regulated peptides relative to the control group originated (Figure 3). This type of analysis reveals the biological significance of the differentially regulated peptides in severe acute pancreatitis. In this evaluation, the following enriched terms were identified at 12 and 24 h after pancreatitis induction: bacterial invasion of epithelial cells, dilated cardiomyopathy, hypertrophic cardiomyopathy, and viral myocarditis (Figure 3). All these terms were even more enriched compared to the control group at the latest time point analyzed. The term regulation of actin cytoskeleton was enriched only in the 24‐h evaluation (Figure 3).

## 4 Discussion

This study reveals dynamic changes in the plasma peptidome during the progression of severe acute pancreatitis in rats. Thus, the dynamic peptidome analysis presented here provides deeper insights into the pathogenesis of the disease [13, 19].

Both protein and peptide expression have already been analyzed in acute pancreatitis; however, peptidomics kinetics during this condition lacks studies in the literature [10, 12, 13, 19, 20]. Severe acute pancreatitis has a dynamic evolution, progressing from a pro‐inflammatory state in the initial phase to immunosuppression later [21]; similarly, the peptidome profile exhibits evolutionary characteristics, as identified in this study.

The highest number of differentially regulated peptides was observed at 12 h. These findings may reflect the severity of the disease at this time point, which presented a 46% mortality rate between 12 and 24 h after pancreatitis induction. It can be inferred that the time of highest alteration in peptide expression may be a marker of prognostic value. In the clinical scenario, a similarly high mortality, up to 30%, is seen among patients with multiple organ dysfunction and infected pancreatic necrosis necessitating invasive procedures [1].

Derivatives of the alpha‐1 microglobulin (A1M) protein were identified 3 h after pancreatitis induction. This protein is a tissue protector due to its antioxidant properties, controlling free radicals in extravascular fluids and promoting tissue repair [22, 23]. The upregulation of these peptides at 6 and 12 h may represent increased degradation of the A1M protein, suggesting an imbalance in the control of the inflammatory response.

The only differentially regulated peptide, showing an increase at 1 h after pancreatitis induction, is derived from the beta component of fibrinogen (FIBB). It is important to highlight that fibrinogen degradation is associated with pro‐inflammation [24]. There is a direct relationship between inflammation and coagulation, in such a way that their interactions are bidirectional.

Peptides derived from cytoskeleton proteins (ACTS and ACTG) are differentially regulated at 12 and 24 h. However, it is observed that peptides derived from ACTS are increased in relation to the control, while those derived from ACTG are decreased, which could be due to protein counter‐regulation, representing cytoskeleton remodeling. Corroborating these findings, Tashiro et al. [25] showed, in an experimental model of pancreatitis, that cytoskeleton disruption occurs 12 h after pancreatitis induction. Yao et al. [26] observed that ACTG1 is increased in sepsis and is associated with a worse prognosis. These same authors blocked ACTG1 with RNA interference, in vitro, and found a decrease in apoptosis, suggesting that this protein may be a promising therapeutic target in sepsis.

Through the identification of differentially regulated peptides, bioinformatic analysis was performed to define the altered pathways and genes, allowing a better understanding of the mechanisms and participation of these peptides in severe acute pancreatitis. The 6‐h time point after pancreatitis induction showed the highest number of signaling terms/pathways, represented by the mTOR, quorum sensing, JAK/STAT, adherens junctions, cell adhesion molecules, and phospholipase D pathways. It can be inferred that this period characterizes the inflammatory phase of the disease, and in subsequent moments, the activation of other pathways implies a predisposition to infection. This is based on the enrichment of pathways related to infection and the regulation of the actin cytoskeleton at 12‐ and 24‐h post‐pancreatitis induction, suggesting that this period was associated with the process of bacterial translocation and pancreatic necrosis infection.

Upon analysis of the protein interaction network with the genes encoding the differentially regulated peptides, it was observed that the actin cytoskeleton regulation pathway was enriched 24 h after pancreatitis induction. Corroborating these findings, Lassout et al. [20] also demonstrated an increase in peptides derived from cytoskeleton proteins in animals subjected to mild acute pancreatitis compared to the control group (27.6% vs. 19.3%). In the bioinformatics analysis, Fan et al. [11] observed increased expression of the beta‐actin gene, suggesting it as a possible biomarker for diagnosis and treatment. Furthermore, they reported that this gene is related to bacterial invasion pathways, which may reinforce the regulation of the cytoskeleton with infection [11].

Infection of pancreatic necrosis occurs late in pancreatitis, usually within 2 weeks of the onset, and with a mortality rate of 10% to 20% [1, 4]. In an experimental model, Foitzik et al. [27] demonstrated pancreatic infection in 33% of the animals 24 h after induction of severe acute pancreatitis, rising to 75% after 48 h. Consistent with these findings, enrichment of the quorum‐sensing pathway, with greater activity at 24 h, and the bacterial invasion of the epithelial cell pathway, after 12 h of pancreatitis induction, were observed. Both pathways and terms were involved in the pathogenesis of bacterial infection, including intercellular communication and cellular invasion [28]. SLiMs aid in understanding the biological functions of peptides through bioinformatics analysis [16]. Upon evaluating the SLiMs of differentially regulated peptides, a greater number of increased pathways/terms was observed after 6 h of pancreatitis induction.

In pancreatitis, the impairment of cell adhesion between pancreatic acinar cells and the endothelium results in altered permeability, compromising the tissue structure of the organ [29]. In this sense, the cell adhesion molecules pathways/terms were shown to be altered between 3 and 12 h, but with the progression of the disease, greater activity of the pathways occurs with more SLiMs involved. This is in agreement with Barbeiro et al. [30], which in an experimental model of severe pancreatitis, observed a decrease in tight junctions in the intestine, with alteration of the intestinal barrier, culminating in bacterial translocation.

The Janus kinase/STAT pathway, which is enriched and started 6 h after pancreatitis induction, and declines at 24 h, is noteworthy. Li et al. [31] also demonstrated that inhibition of the JAK2/STAT3 pathway resulted in lower pro‐inflammatory activity, and increased expression after 18 h of pancreatitis induction. In a cellular model of pancreatitis, Chen et al. [32] observed early expression of this pathway and correlation with transcription of pro‐inflammatory cytokines. In an experimental model of sepsis, Hui et al. [33] found greater activity of JAK/STAT, and inhibition of this pathway resulted in greater survival. Loss or mutation of the JAK/STAT pathway is associated with various diseases, such as cancer and autoimmune conditions, and inhibition or stimulation of this pathway may have an impact on disease treatment [34].

Another pathway also enriched at 6 h was the mTOR. This pathway modulates important cellular events, notably protein synthesis, actin dynamics of the cytoskeleton, cell proliferation, and autophagy. mTOR is interconnected with the AKT pathway, as it regulates and is positively regulated by this pathway. Huangfu et al. [35] demonstrated mTOR/AKT activation in experimental acute pancreatitis and that early inhibition of this pathway results in less oxidative stress, in addition to reducing pancreatic injury by stimulating autophagy. Similarly, Chen et al. [36] analyzed AKT knockout mice with mild acute pancreatitis and observed that these animals had less inflammatory infiltrate in the pancreas. On the other hand, Hu et al. [37] showed that late inhibition of mTOR has deleterious effects, denoting the dynamics of the inflammatory response, which shows the relevance of the timing and type of intervention to promote disease control. This is reflected in severe acute pancreatitis, in which attenuation of the inflammatory response in the initial phase may have beneficial effects, but in the late phase would imply necrosis, infection, and mortality [21].

Based on the results, it can be concluded that peptides related to alpha‐1 microglobulin peptides may be an early marker of severity prognosis, considering that it is increased from 3 h and in subsequent periods. The alterations in cytoskeleton proteins reflect the impairment of the pancreatic parenchyma, which predisposes to the occurrence of infection. In the bioinformatics analysis, it is observed that the infection pathways, quorum sensing, and bacterial invasion of epithelial cells increase with the course of the disease, suggesting that the late altered peptides may be markers of infected pancreatic necrosis.

A limitation of the present study is that pathway enrichment analyses were based on a small number of source proteins, particularly at early time points. Therefore, these results should be interpreted cautiously as hypothesis‐generating rather than definitive evidence of pathway activation. Another shortcoming is the absence of a sham‐operated control group. Although the taurocholate model reliably induces pancreatitis, it is possible that surgical stress or anesthesia contributed to some peptide alterations, particularly those related to immune and cytoskeleton pathways.

Defining the dynamics of the peptidome profile in severe acute pancreatitis can be a tool to early recognize which human subjects will develop infection. Furthermore, with the aid of bioinformatics, understanding the pathways in the evolution of pancreatic necrosis may be an alternative for specific therapeutic interventions at different times.

## Funding

E.S.F. acknowledges the São Paulo Research Foundation (FAPESP, grant 2016/04000‐3) and the Brazilian National Research Council (CNPq; grant PQ 303096/2021‐7). L.K.I. acknowledges Cepid/CeTICS from Butantan Institute (FAPESP grant 2013/07467‐1).

## Conflicts of Interest

The authors have declared no conflict of interest

## Data Availability

The LC‐MS/MS raw data files are deposited at the Center for Computational Mass Spectrometry of the University of California, San Diego, MassIVE repository. They can be downloaded from: ftp://www.massive‐ftp.ucsd.edu/v11/MSV000099750/.
